# The Antihelmintic Drug Pyrvinium Pamoate Targets Aggressive Breast Cancer

**DOI:** 10.1371/journal.pone.0071508

**Published:** 2013-08-27

**Authors:** Wei Xu, Lara Lacerda, Bisrat G. Debeb, Rachel L. Atkinson, Travis N. Solley, Li Li, Darren Orton, John S. McMurray, Brian I. Hang, Ethan Lee, Ann H. Klopp, Naoto T. Ueno, James M. Reuben, Savitri Krishnamurthy, Wendy A. Woodward

**Affiliations:** 1 Division of Radiation Oncology, The University of Texas MD Anderson Cancer Center, Houston, Texas, United States of America; 2 StemSynergy Therapeutics, Inc., Lauderdale by the Sea, Florida, United States of America; 3 Department of Experimental Therapeutics, The University of Texas MD Anderson Cancer Center, Houston, Texas, United States of America; 4 Department of Cell and Developmental Biology, Vanderbilt Ingram Cancer Center, Vanderbilt University Medical Center, Nashville, Tennessee, United States of America; 5 Department of Breast Medical Oncology, The University of Texas MD Anderson Cancer Center, Houston, Texas, United States of America; 6 Department of Hematopathology, The University of Texas MD Anderson Cancer Center, Houston, Texas, United States of America; 7 Division of Pathology, The University of Texas MD Anderson Cancer Center, Houston, Texas, United States of America; University of South Alabama, United States of America

## Abstract

WNT signaling plays a key role in the self-renewal of tumor initiation cells (TICs). In this study, we used pyrvinium pamoate (PP), an FDA-approved antihelmintic drug that inhibits WNT signaling, to test whether pharmacologic inhibition of WNT signaling can specifically target TICs of aggressive breast cancer cells. SUM-149, an inflammatory breast cancer cell line, and SUM-159, a metaplastic basal-type breast cancer cell line, were used in these studies. We found that PP inhibited primary and secondary mammosphere formation of cancer cells at nanomolar concentrations, at least 10 times less than the dose needed to have a toxic effect on cancer cells. A comparable mammosphere formation IC50 dose to that observed in cancer cell lines was obtained using malignant pleural effusion samples from patients with IBC. A decrease in activity of the TIC surrogate aldehyde dehydrogenase was observed in PP-treated cells, and inhibition of WNT signaling by PP was associated with down-regulation of a panel of markers associated with epithelial-mesenchymal transition. *In vivo*, intratumoral injection was associated with tumor necrosis, and intraperitoneal injection into mice with tumor xenografts caused significant tumor growth delay and a trend toward decreased lung metastasis. In *in vitro* mammosphere-based and monolayer-based clonogenic assays, we found that PP radiosensitized cells in monolayer culture but not mammosphere culture. These findings suggest WNT signaling inhibition may be a feasible strategy for targeting aggressive breast cancer. Investigation and modification of the bioavailability and toxicity profile of systemic PP are warranted.

## Introduction

Of the cells that make up a tumor's bulk, a small population is purported to consist of tumor initiation cells (TICs), which have self-renewing and multipotent properties that lead to treatment resistance and recurrence. Accumulating evidence suggests that cells bearing functional and cell surface markers that correlate to tumor initiation are regulated by developmental pathways that are critical to normal stem cell survival, including Sonic hedgehog signaling [1], Notch signaling [2], [3], [4], and WNT signaling [5], [6]. These pathways are attractive targets for potentially eliminating the TIC subpopulation.

WNT signaling was first identified in investigation of the embryonic lethal mutations that alter the pattern of the *Drosophila* embryonic cuticle [7], [8]. Later on, it was found to play a key role in the development of vertebrates [9]. It was independently identified by Nusse and Varmus in a screen using MMTV for proto-oncogenes [10] and was later shown to be involved in mesoderm induction [11]. Ultimately, more and more findings suggested that the function of the WNT signaling pathway reaches far beyond embryonic development. Abnormal activation of WNT signaling was found in multiple cancers, most notably the APC mutation in 80% of all nonhereditary colon cancers [12], [13]. In human breast cancer, even though overt mutations of WNT signaling pathway members are rarely found, it has been demonstrated that several members of this pathway were changed in other ways [14]. Nuclear β-catenin, one of the key components in WNT signaling, was found to overexpress in 40–60% of human breast cancer [15], [16], [17]. In another report, *Dvl1*, another key component of WNT signaling, was shown to be over-expressed in some breast cancers [18]. Moreover, LRP6, the co-receptor for WNT, was found to be up-regulated in triple-negative breast cancer specifically [19]. The WNT signaling pathway was also found to play an important role in human embryonic stem cell self-renewal [20], [21], as well as in the self-renewal of mammary gland stem cells [22]. Considering the signaling pathway similarity between cancer stem cells and normal stem cells [23], it is not surprising that the WNT signaling pathway also plays a key role in the self-renewal of breast cancer TICs [6], [24].

Several studies have suggested that compared with differentiated cancer cells, TICs are more resistant to conventional cancer therapies. In a recent study, it was found that conventional chemotherapies, such as docetaxel or doxorubicin plus cyclophosphamide, increased the percentage of the CD44^high^CD24^low^ subpopulation, a surrogate for human breast cancer TICs, in pre- vs. post-treatment patient samples [25], [26]. Similarly, in several solid tumor types, radiation has been shown to enrich for TICs by selectively targeting the non-TIC population [24], [27], [28]. Since TICs are attractive targets for cancer therapies, efforts are under way to identify drugs that target this population. Drugs that have been reported to inhibit TICs include salinomycin, an anticoccidiostat ionophore [29]; repertaxin, a small molecule inhibitor of CXCR1 [30]; sulforaphane, a dietary component of broccoli and broccoli sprouts that inhibits WNT signaling [31]; and perifosine, a small molecule inhibitor of AKT signaling [5], [6].

Pyrvinium pamoate (PP) is an oral FDA-approved antihelmintic drug that was first described in the 1950s [32]. When taken orally, it is safe even at high doses, but systemic absorption of PP from the gut is minimal [33]. In a recent study by Thorne et al [34], β-catenin signaling was shown to be potently targeted by PP. In their study, they found that the stability of β-catenin was decreased by this drug through the activation of casein kinase 1α. Although additional clinical studies and potential structural modifications would be needed to use this drug in the treatment of disease outside the lumen of the gut, its activity makes it a potentially important agent for targeting WNT signaling.

In the study reported herein, we used PP to inhibit WNT signaling and examined its impact on TICs of aggressive breast cancers. *In vitro*, we confirmed that PP decreased the steady level of β-catenin expression. PP inhibited proliferation with a half maximal inhibitory concentration (IC50) at least 10 times higher than the dose that inhibited primary and secondary mammosphere formation. PP was also shown to decrease the aldehyde dehydrogenase (ALDH)-positive cell population and the levels of multiple epithelial-mesenchymal transition (EMT) markers. *In vivo*, PP delayed tumor growth [in mice?] and caused marked tumor necrosis. *Ex vivo*, PP decreased the mammosphere formation of breast cancer cells from fresh pleural effusion fluid with an IC50 value similar to that of breast cancer cell lines. We concluded that WNT signaling and TICs are potentially inhibited by PP and thus further investigation of this agent is warranted.

## Materials and Methods

### Cell culture

SUM-149 inflammatory breast cancer (IBC) cells and SUM-159 metaplastic basal-type breast cancer cells were purchased from Asterand (Detroit, MI). They were cultured in Ham's F-12 media supplemented with 10% fetal bovine serum (FBS), 1 µg/ml hydrocortisone, 5 µg/ml insulin, and an antibiotic-antimycotic (Invitrogen, Grand Island, NY).

Fresh cells were obtained from pleural fluid from patients with IBC under a protocol (LAB06-0726). This protocol has been approved by the institutional review board from a patient with inflammatory breast cancer (Protocol No. LAB06-0726). This protocol was specifically reviewed and approved by University of Texas MD Anderson ethics committee. According to this protocol, patients provide written informed consents before sample were taken. Breast cancer cells were isolated from the pleural fluid as follows. Briefly, the pleural fluid was centrifuged at 400 *g* for 30 min. The pellets were suspended in phosphate-buffered saline (PBS)/Hank's balanced salt solution (HBSS) and filtered through a 40-µM cell strainer (Invitrogen, Grand Island, NY). The suspended cells were added to the top of 12.5 [ml?] Ficoll Histopaque solution (Sigma, St. Louis, MO), and the mixture was spun at 2000 rpm for 30 min. The centrifuged cells were washed with PBS 3 times and suspended in PBS prior to the mammosphere formation and Aldefluor assays described below.

### Analysis of β-catenin signaling activity

We investigated WNT signaling activation in live cells using a construct that contains TOP (a WNT response promoter) [41] followed by the gene for green fluorescent protein (GFP). 7-TGP is a lentivirus-based construct in which 7 TOP promoters control the expression of GFP [35]. The construct was requested from Addgene, and lentivirus was made as previously described [36]. Then, SUM-159 cells were transduced with virus and positive cells were selected using puromycin resistance. Transduced cells and untransduced cells were treated with PP at indicated concentrations for 96 hours, and GFP expression was analyzed by flow cytometry.

### MTS assay

SUM-149 and SUM-159 cells were seeded in 96-well microplates at a density of 5,000, 10,000, and 20,000 per well. Cells were treated with indicated doses of PP. In 96 hours, cell viability was assessed by a 3-(4,5-dimethylthiazol-2-yl)-5-(3-carboxymethoxyphenyl)-2-(4-sulfophenyl)-2H-tetrazolium (MTS) assay (Promega, Madison, WI) according to the manufacturer's instructions. In this assay, the number of living cells is directly proportional to the absorbance at 490 nm of a formazan product, which is reduced from MTS by living cells.

### Counting cells by flow cytometry

SUM-159 cells were seeded in 6-well plates at a density of 100,000 per well. Cells were treated with indicated doses of PP. In 96 hours, cells were trypnized by 200 µl trypsin followed by adding 800 µl culture media. 20 µl CountBright absolute counting beads (Invitrogen, Carlsbad, CA) were added to each dose and flow cytometry was carried out to count the live cells and beads. Propidium Iodine was used to exclude the dead cells. In this assay, the number of living cells is calculated by the ratio between events and beads.

### Primary and secondary mammosphere assays

The effect of PP on mammosphere formation, a characteristic of mammary stem/progenitor cells, was assessed. For the primary mammosphere assay, SUM-149 and SUM-159 cells from monolayer culture were first dispersed into single cells. For the secondary mammosphere assay, cells from monolayer culture were cultured as spheres in a 10-cm ultra-low-attachment dish for 96 hours with the indicated doses of PP, and then those mammospheres were collected and dispersed as single cells. For both mammosphere assays, single cells were then grown in serum-free, growth factor-enriched conditions in low-attachment plates [37]. Specifically, cells were grown in 6-well ultra-low-attachment plates in serum-free minimum essential medium (MEM) supplemented with 20 ng/ml basic fibroblast growth factor, 20 ng/ml epidermal growth factor, 1 µg/ml hydrocortisone, 5 µg/ml insulin, and 2% B27 (all from Invitrogen). Cells were plated at 20,000 cells/ml unless specified otherwise. Suspension cultures were incubated for 7 days. After staining with 3-(4,5-dimethylthiazol-2-yl)-2,5-diphenyltetrazolium bromide (MTT) (Sigma, St. Louis, MO) in order to increase the contrast and allow automated detection of viable spheres, colonies were counted with an automated 3D colony counter (Oxford Optronix, Oxford, UK).

### Aldefluor assay

The Aldefluor assay was carried out according to the manufacturer's guidelines (StemCell Technologies, Vancouver, Canada). Briefly, single cells obtained from SUM-159 cell cultures, or cells purified from fresh pleural effusion fluid, were treated with indicated doses of PP or DMSO for 96 hours. Cell cultures or xenograft tumors were incubated in an Aldefluor assay buffer containing an ALDH substrate for 30 min at 37°C. As a negative control, half of the cells from each sample were incubated under the same conditions in the presence of the ALDH inhibitor diethylaminobenzaldehyde (DEAB). Flow cytometry was used to measure the ALDH-positive cell population.

### Western blotting

Cells grown as described above were incubated for 4 days in the indicated concentrations of PP. Cells were then lysed in 1× RIPA lysis buffer, and Western blots were performed as previously described [38]. Briefly, aliquots of the supernatants containing 50 µg protein were electrophoresed on 4–20% gradient sodium dodecyl sulfate-polyacrylamide gels (Invitrogen, Grand Island, NY) and transferred to polyvinylidene fluoride membranes (Bio-Rad Laboratories, Hercules, CA). The membranes were incubated in 5% nonfat milk for 1 hour at room temperature and then incubated at 4°C overnight with the following antibodies: N-cadherin, vimentin (BD Biosciences, San Jose, CA), dephospho-β-catenin, β-catenin, Slug, and GAPDH (Cell Signaling Technology, Danvers, MA). After incubation with the secondary antibody (Santa Cruz Biotechnology, Santa Cruz, CA), the membranes were washed and immunoreactivity was detected by enhanced chemiluminescence. GAPDH served as a loading control.

### Breast cancer xenografts in mice

For our *in vivo* experiment, 3- to 5-week-old female SCID/Beige mice (Harlan, Indianapolis, IN) or Swiss nude mice were housed and used in accordance with institutional guidelines of MD Anderson Cancer Center under an Institutional Animal Care and Use Committee (IACUC)-approved protocol (ACUF 07-08-07213). The UTMDACC's animal care and use program has been fully accredited by the Association for the Assessment and Accreditation of Laboratory Animal Care International (AAALAC). The institutional IACUC has specifically reviewed and approved this protocol. Mice were anesthetized with isoflurane (2.5%), and fur at the surgical site was removed. Xenografts were created using cells or tumor chunks as indicated. The inguinal #4 glands were cleared of mammary epithelium and cells or tumor chunks were implanted into the cleared inguinal fat pads. Transplants were allowed to grow out for 7–20 weeks.

In the case of intraperitoneal (IP) delivery, mice whose tumors reached volumes of 100 mm^3^ were treated with different regimens as described in the Results section. In the case of intratumoral (IT) delivery, tumors with volumes of 300 mm^3^ were treated with DMSO (vehicle, 50 µl) or PP dissolved in DMSO (0.1 µg, 50 µl) administered IT twice a week for 3 weeks. During this period, tumor size was measured by calipers and tumor growth was plotted. At the end of the experiment, mice were euthanized and a portion of the tumor and tissue sections from the liver, lungs, and spleen were formalin fixed for further pathologic examination. At the same time, part of each tumor was digested to measure the ALDH-positive population in the tumor.

For pathologic evaluation, tumors and tissues fixed in formalin were embedded in paraffin, sectioned, and stained with hematoxylin and eosin. All staining was performed with standard protocols and analyzed by a pathologist (Savitri Krishnamurthy) who specializes in breast cancer. Percentage of tumor necrosis was evaluated.

### *In vivo* imaging of xenograft tumors and WNT signaling

7-TFP, a lentivirus-based construct containing the TOP WNT-responsive promoter followed by the luciferase gene [39], was used to image the tumor and visualize WNT signaling *in vivo*. SUM-159 cells were transduced with 7-TFP and transplanted into 3-week-old female Swiss nude mice as described above. After 3 weeks, *in vivo* optical imaging was performed weekly using a Xenogen IVIS bioluminescence/fluorescence optical imaging system (Caliper Life Science, Hopkinton, MA). The procedure was as follows. Five minutes prior to imaging, each mouse was given a 100-µl IP injection of D-luciferin (Promega, Madison, WI) at a dose of 125 mg/kg. General anesthesia was then induced with 5% isoflurane (IsoSol; Medeva Pharmaceuticals, Inc., Rochester, NY), and the mouse was placed in a light-tight heated chamber; anesthesia was continued during the procedure with 2% isoflurane introduced via a nose cone. The imaging system consists of a cooled, back-thinned, charge-coupled device (CCD) camera that captures both a visible light photograph of the animal taken with light-emitting diodes and a luminescent image. After acquiring photographic images of each mouse, anterior luminescent images were acquired with 1-min exposure times. The resulting gray-scale photographic and pseudocolor luminescent images were automatically superimposed so that identification of any optical signal with its location on the mouse was facilitated. Optical images were displayed and analyzed with IVIS Living Image (Caliper Life Sciences, Hopkinton, MA) software.

### Clonogenic assays

SUM-149 cells in monolayer culture were treated with the indicated doses of PP for 4 days followed by the indicated doses of radiation. Immediately after radiation, cells were trypsinized, counted, and seeded into mammosphere culture or regular adherent culture. In 7–14 days, clones consisting of a minimum of 50 cells were counted, and numbers were plotted using an in-house macro to generate survival curves and the surviving fraction in SigmaPlot, version 8.0 (Systat Software, San Jose, CA). Data were normalized to the plating efficiency to assess radiosensitization independent of the action of the drug alone.

### Statistics

Excel 2007 was used for all statistical analysis. All continuous data were averaged and the means compared with the Student two-sided *t*-test; P<0.05 was considered significant. Error bars on graphs represent standard deviation. Tumor growth measurements were plotted and compared using an in-house program in Excel 2007.

## Results

### PP inhibited WNT signaling

**Figure 1:**
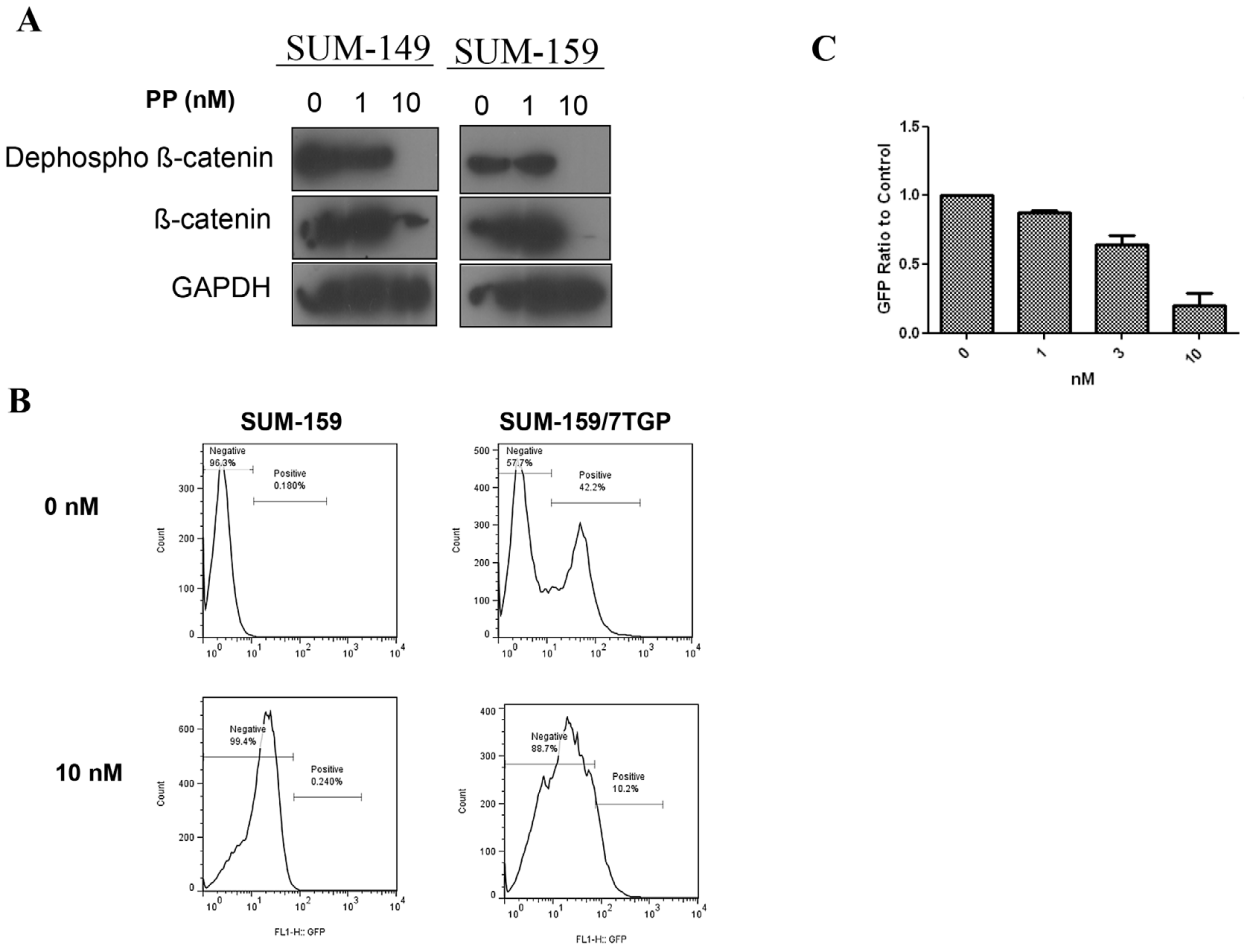
PP targeting of the WNT/β-catenin pathway. (A) PP decreased the protein levels of nonphospho-β-catenin, an active form of β-catenin, and β-catenin in SUM-149 and SUM-159 cell lines. Data shown are representative of three independent experiments. (B) PP also decreased the GFP-positive population in SUM-159 cells transduced with a TOP-GFP construct. (C) Summary of three independent GFP-detection experiments.

To confirm this activity in breast cancer cell lines, we examined whether β-catenin and non-phosphorylated β-catenin (non-phospho-β-catenin) are downregulated by PP in human breast cancer cells. Phosphorylated β-catenin is targeted for degradation, and non-phospho-β-catenin is the active form of β-catenin. As shown in Fig. 1A, PP decreased the protein levels of β-catenin and non-phospho-β-catenin significantly in triple-negative (negative for estrogen receptor, progesterone receptor, and HER-2-neu amplification) SUM-149 and SUM-159 cells in a dose-dependent manner.

WNT signaling activation can be further detected using a construct that contains TOP (a WNT response promoter) [40] followed by the gene for luciferase or GFP. We used a lentivirus-based construct containing the WNT response promoter TOP followed by the GFP gene [39] to detect WNT activation in cancer cells. Because PP has autofluorescence, untransfected SUM-159 cells were used as a negative control (Fig. 1B). As shown in Fig. 1C, PP decreased WNT signaling in SUM-159 cells in a dose-dependent manner. These results are consistent with previous findings of WNT inhibition with this agent [34].

### The toxic effect of PP on breast cancer cells

**Figure 2:**
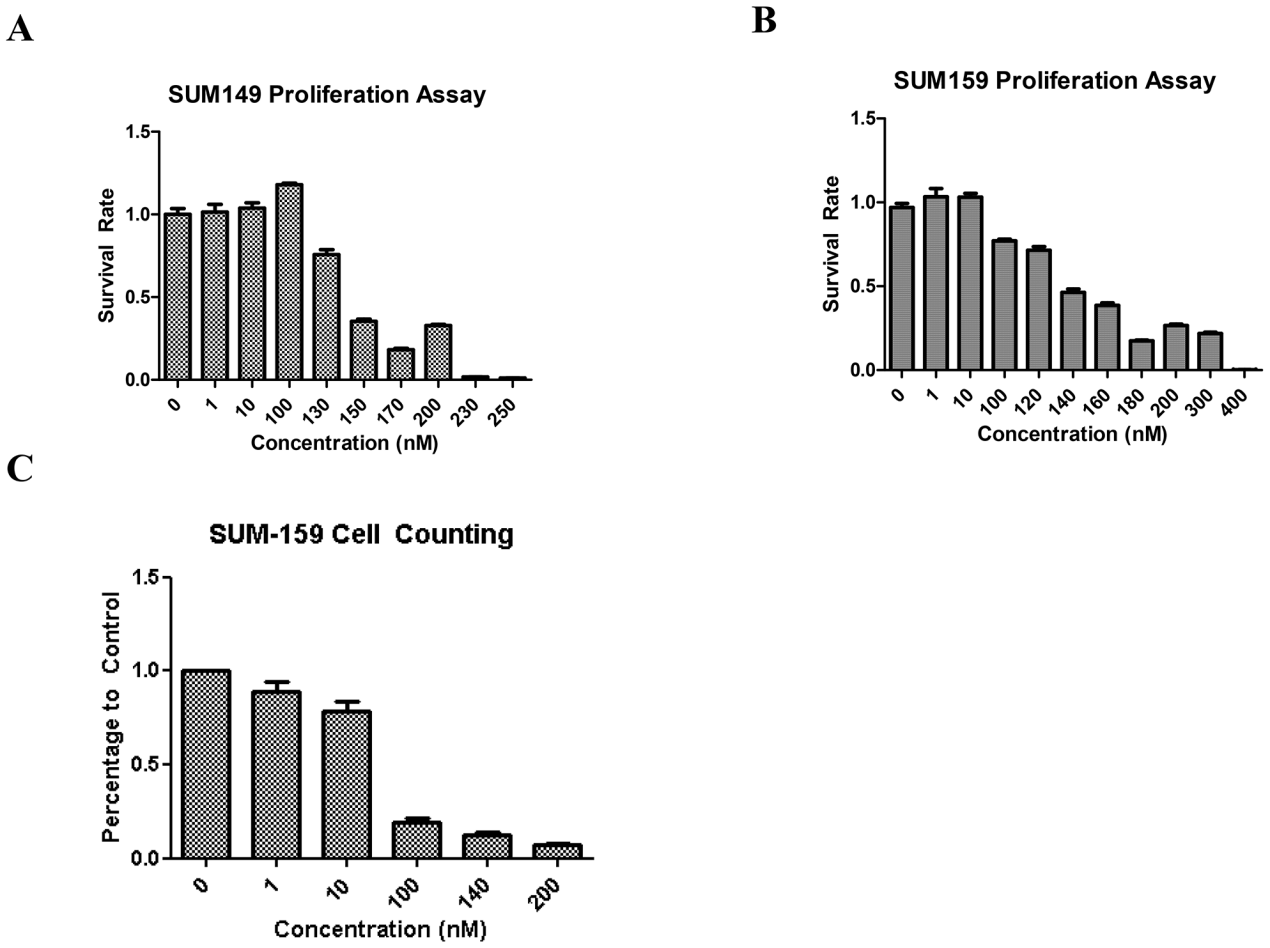
The toxic effects of PP on breast cancer cells. SUM-149 and SUM-159 were treated with increasing concentrations of PP for 96 hours. The toxic effect of PP was measured by MTS assay (A, B) or cell counting by flow cytometry (C). The data shown are representative or summary of three independent experiments.

We first evaluated the toxic effects of PP in SUM-149 and SUM-159 human breast cancer cell lines by MTS assay. Cells were treated with increasing concentrations of PP for 96 hours, and the ratios of viable cells of treatment cultures relative to control cultures are plotted in Fig. 2A and Fig. 2B. MTS activity decreased as the concentration of PP increased, with IC50 values of 170.0±42.2 nM for SUM-149 and 163.3±32.1 nM for SUM-159. MTS activity may be reduced secondary to decreased survival or decreased proliferation. Given PP may inhibit NADPH oxidoreductase activity in cells we examined absolute viability using flow cytometry based absolute cell counting (Fig. 2C). In this technology, flow detectable beads are added in a known ratio relative to the cell suspension volume. After flow cytometry, absolute number of living cells was calculated using the number of live cells, the number of detected beads and the known ratio of beads to cell suspension. As shown in the Fig. 2C, the IC50 value for 159 is around 60 nM, which is lower than that from MTS assay. This indicated the interference of PP in the MTS assay.

### PP inhibited breast TIC surrogates (mammospheres) *in vitro*

**Figure 3:**
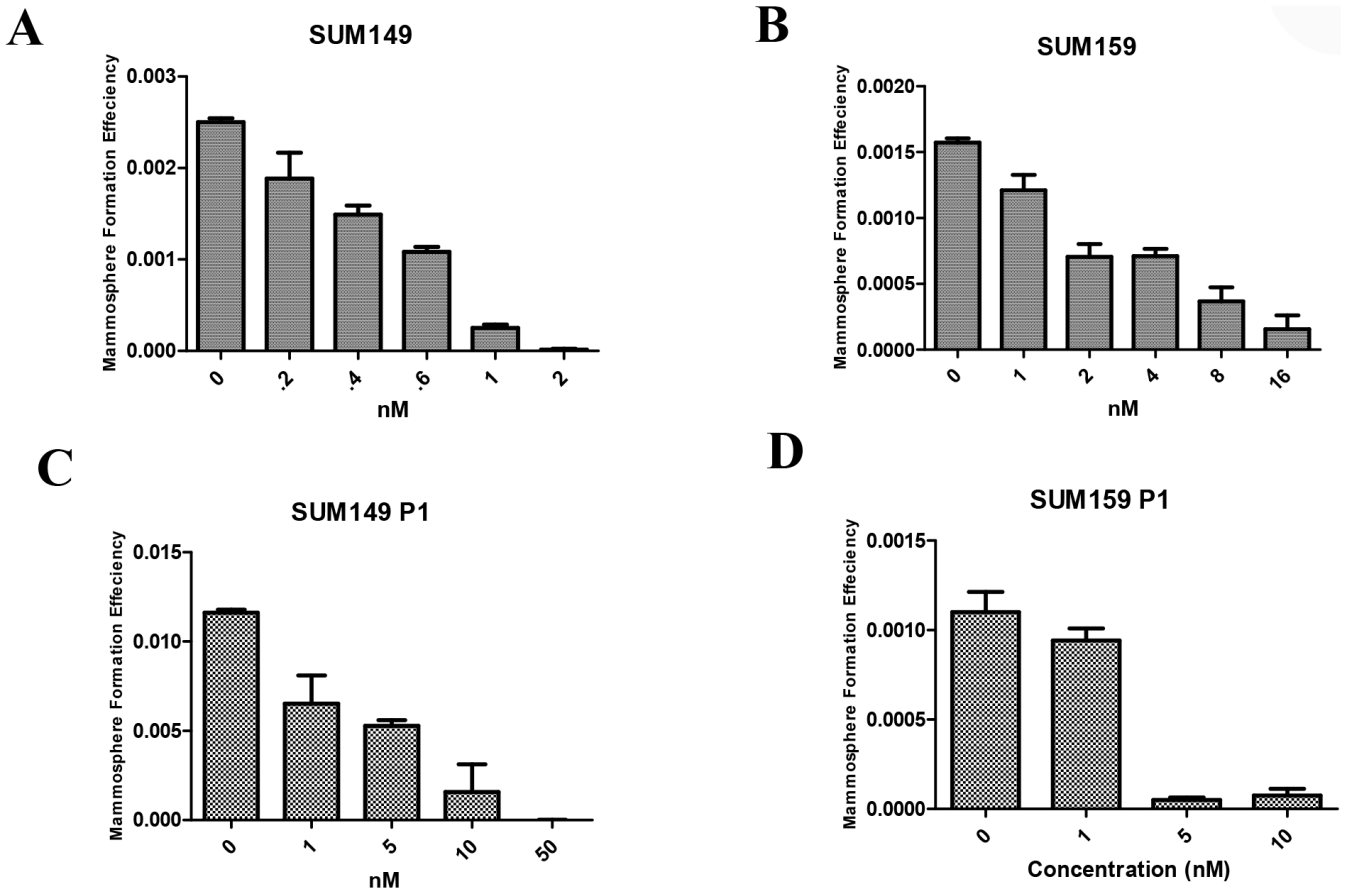
Inhibitory effect of PP on mammosphere formation. (A, B) Primary mammosphere formation. SUM-149 (A) and SUM-159 (B) cells were incubated with indicated doses of PP or DMSO in mammosphere formation media for 7 days. PP treatment reduced the number of primary mammospheres in a dose-dependent manner. (C, D) Secondary mammosphere formation. SUM-149 (C) and SUM-159 (D) cells were grown in mammosphere formation media and treated with indicated doses of PP for 4 days. Then primary spheres were trypsinized and seeded as secondary mammospheres. [Add sentence summarizing effect of PP.] The results shown are representative of three independent experiments.

It has been shown that mammary stem/progenitor cells are enriched in nonadherent spherical clonogens of cells, termed mammospheres. These cells are capable of yielding secondary spheres and differentiating along multiple lineages [37]. To evaluate whether PP can suppress the formation of mammospheres *in vitro*, we exposed primary SUM-149 and SUM-159 spheres to varying concentrations of PP. As shown in Fig. 3A and 3B, IC50 values for SUM-149 and SUM-159 were 0.44±0.11 nM and 2.70±1.21 nM, respectively. We tested the effect of PP on the self-renewal of breast cancer TIC surrogates through the use of secondary mammosphere assays. As shown in Fig. 3C and 3D, the IC50 doses of PP for inhibiting secondary mammosphere formation were 7.50±3.53 nM for SUM-149 and 2.36±1.34 nM for SUM-159. It is worth noting that the IC50 doses of PP for inhibiting primary and secondary mammosphere formation were 10 times lower than the IC50 doses for toxicity in both cell lines.

### PP decreased the ALDH-positive population in breast cancer cells

**Figure 4:**
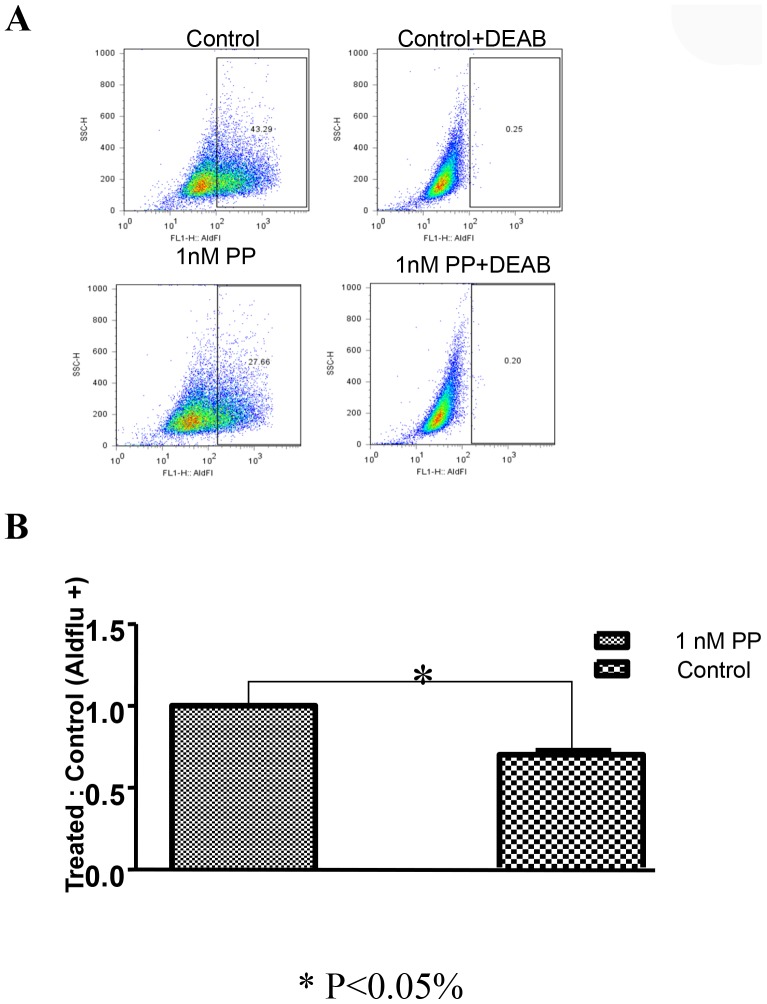
PP decreased the ALDH-positive cell population. SUM-159 cells were treated with PP (1 nM) or DMSO for 96 hours and subjected to an Aldefluor assay and flow cytometry analysis. (A) A set of representative flow cytometry dot plots. DEAB served as a negative control. (B) PP decreased the percentage of ALDH-positive cells. The data shown are the means of three independent experiments.

In aggressive breast cancer cell lines, a cell population with high ALDH activity as assessed by the Aldefluor assay has been shown to be enriched in tumorigenic stem/progenitor cells and is prognostic in IBC [41]. This cell population is capable of self-renewal and generating tumors resembling the parental tumor [41]. Because SUM-159 has a relatively high percentage of ALDH-positive cells, we selected SUM-159 to examine whether PP inhibits tumor-initiating ALDH-positive cells *in vitro*. Representative flow cytometry dot plots are presented in Fig. 4A. As shown in Fig. 4B, 1 nM PP decreased the ALDH-positive population of SUM-159 cells by over 35% (P<0.05). These data showed that PP inhibited the ALDH-positive cells at similar concentrations to those inhibiting mammosphere formation and at concentrations at least 10-fold lower than those that caused toxicity effects on the cancer cells as determined by MTS assay.

### PP effects *in vivo* (xenograft model in mice)

We next tested PP in a mouse model. Tumors were established by injecting SUM-159 cells into the mammary fat pads of SCID/Beige mice. Given the reported limited bioavailability of PP [33], we initially delivered PP through IT injection. Once tumors grew to the size of 300 mm^3^, either vehicle (DMSO) or 0.1 µg PP was injected into tumor directly twice a week for 3 weeks, after which the mice were euthanized. PP delayed tumor growth (Fig. S1A), caused tumor necrosis (Fig. S1B), and decreased the ALDH-positive population (Fig. S1C). The tumor necrosis was confirmed by histologic analysis (Fig. S1D). Three of 5 mice in the treatment group had necrosis, while none of the 3 mice in the control group had necrosis (Fig. S1E). Although there was a delay in tumor growth in the treated animals, potentially due to limitations of caliper measurements after massive necrosis (tumor base measurements were largely unchanged; see Fig. S1B) and the small number of mice, the P values for tumor growth delay and percentage of necrosis were non-significant.

**Figure 5:**
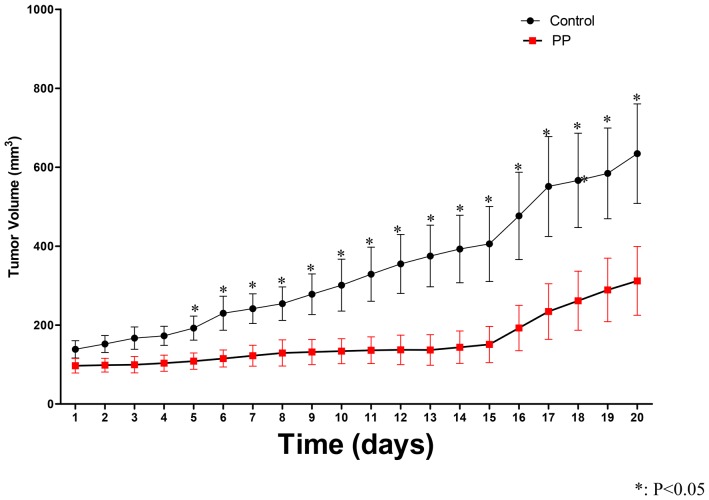
PP inhibited tumor growth *in vivo* when it was administered intraperitoneally. SUM-159 cells were injected into the mammary fat pads of SCID/Beige mice. Once tumors grew to approximately 100 mm^3^, either vehicle (DMSO/saline 50%/50%) or PP was injected IP three times per week. The dose of PP was scaled up according to the following regimen: 1^st^ dose: 0.1 mg/kg; 2^nd^ dose: 0.2 mg/kg; 3^rd^ dose: 0.4 mg/kg; 4^th^ dose: 0.6 mg/kg; 5^th^ dose: 0.8 mg/kg; and 6^th^ dose: 1.0 mg/kg. After the 6^th^ injection, the dose was kept at 1 mg/kg. PP delayed tumor growth beginning at 4 days of the initial treatment. There were 11 mice in the treatment group and 10 mice in the control group. The Student *t*-test was used to calculate P value. Error bars indicate standard deviation.

To assess systemic efficacy and toxicity, we tested IP administration of the SUM-159 xenograft model cells in SCID/Beige mice. Two million SUM-159 cells were transplanted into the cleared mammary fat pads of SCID/Beige mice. Only one gland per mouse was implanted. Once tumors reached approximately 100 mm^3^, specified doses of PP dissolved in 100 µl DMSO/saline (50%/50%) or 100 µl DMSO/saline (50%/50%) alone were injected IP 3 times a week. The dose was scaled up from 0.1 mg/kg to 1 mg/kg as described in Fig. 5. We chose this regimen after a preliminary experiment in which 5 of 9 mice died after 2 injections of PP at 1 mg/kg while 7 mice injected with the same volume of vehicle showed no signs of toxicity. When the tumor volume reached 1000 mm^3^, mice were euthanized and tumors and normal tissues were excised. Part of each tumor and normal tissue was fixed in a formalin solution and subjected to histologic examination. As shown in Fig. 5, PP caused significant tumor delay with near complete response in 5 of 11 treated mice. This observation potentially highlights the issue of bioavailability but deserves further study. As expected, histological examination of tumor tissue revealed no significant histologic differences between treated and untreated tumors.

### PP decreased WNT signaling *in vivo*

Using lentivirus-based construct 7-TFP, in which TOP promoter is followed by a luciferase gene [39], we confirmed that IP treatment with PP inhibited WNT signaling *in vivo*. SUM-159 cells transduced with the construct were transplanted into Swiss nude mice. In a proof-of-principle experiment, we observed that luciferase signal was detected in tumors in the transplanted mammary glands and that this signal was diminished by PP treatment (Control N = 7, PP N = 9 ; Fig. S2A). Luciferase signal is quantified in Fig. S2B. In this figure, Y-axis are the ratio between the signal after treatment and beginning at treatment. Luciferase signal was lower in the treatment group but no significance was achieved due to the small number of mice.

### PP decreased lung metastasis in a mouse Tumor 505 model

To test the effect of PP on tumor metastasis, a rapidly metastatic murine model, Tumor505, was used. Tumor 505 is a serially propagated tumor from a transgenic mouse model in which Smoothened, the key component of the Sonic hedgehog (Shh) pathway, was constitutively activated in the mouse mammary gland [42]. Murine Tumor505 is very aggressive, and spontaneous metastases happen before tumor volume reaches the limits for acceptable animal use. Interestingly, decreased expression of the downstream target of Shh signaling, Gli, has been associated with pathologic complete response to chemotherapy in IBC patients (N = 65, P = 0.002; personal communication, World IBC Consortium, manuscript submitted). Tumor505 chunks were transplanted into the cleared mammary fat pads of SCID mice. When tumors reached 100 mm^3^, mice were treated with 1 mg/kg PP or vehicle through IP administration. After 3 weeks of treatment, mice were euthanized. Lung tissue was stained with hematoxylin and eosin, and the ratio between the lung metastasis area and total lung area observed was estimated by a board-certified pathologist. Consistent with the hypothesis that TIC-targeted therapy would reduce metastasis, there was a trend toward a significant difference in lung metastasis area between the control and treatment groups (P = 0.055; Fig. S3A and B).

### PP decreased the TICs in primary breast cancer cells from pleural effusion fluid

**Figure 6:**
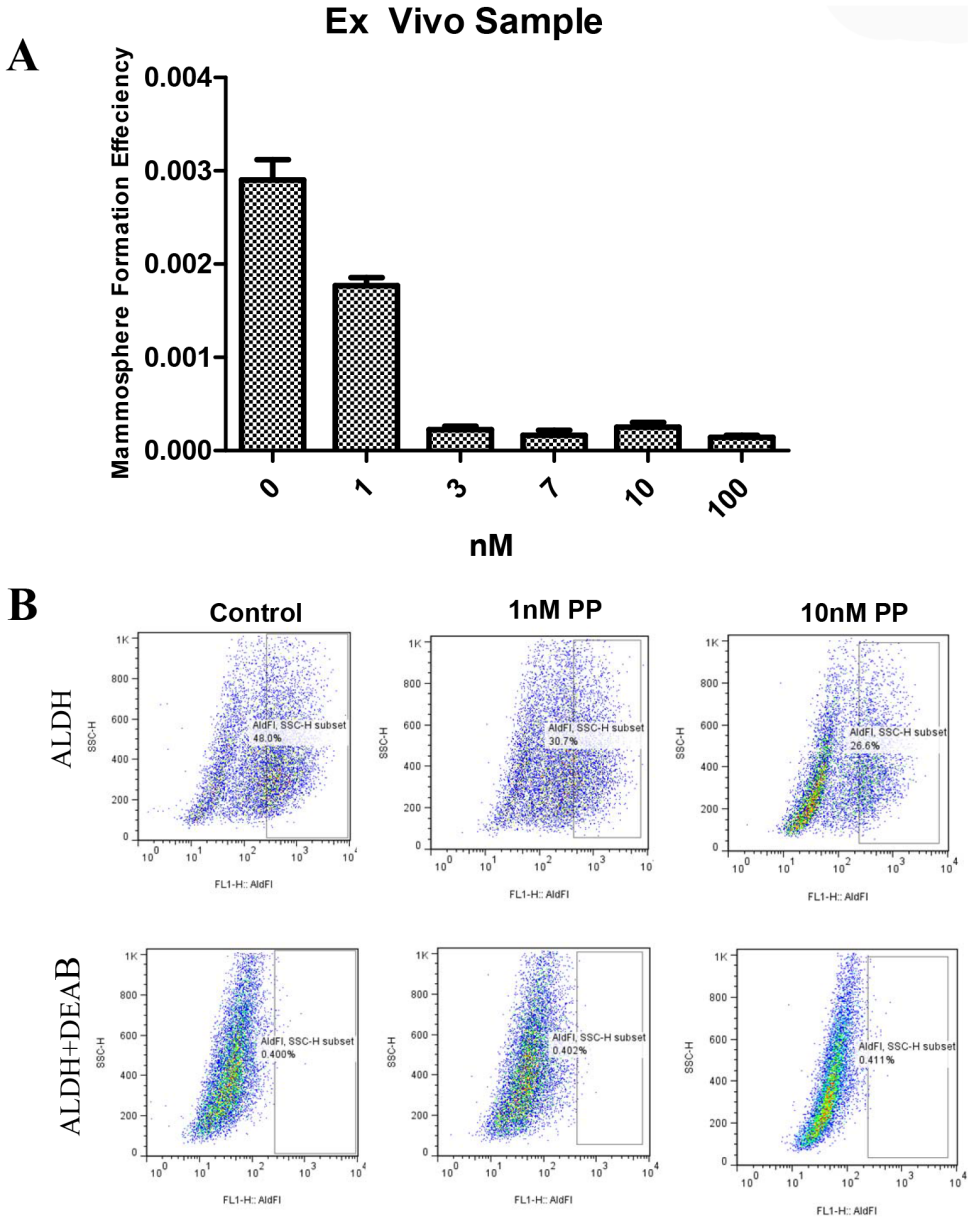
PP inhibited mammosphere formation by primary breast cancer cells. Cancer cells were purified from pleural effusion samples as described. The cells were seeded at 40,000/ml in mammosphere formation media. (A) PP treatment reduced the number of primary mammospheres in a dose-dependent manner. (B) Flow cytometry revealed a decrease in ALDH-positive activity after PP treatment.

We also used cancer cells collected from fresh pleural effusions in patients with breast cancer to test whether PP decreases the TICs in primary breast cancer cells. As shown in Fig. 6A, PP decreased the mammosphere formation of cells from a fresh patient sample at an IC50 similar to that of SUM-159. As seen in Fig. 6B, PP decreased the ALDH-positive population in the primary patient sample.

### PP decreased EMT-related protein expression *in vitro*

**Figure 7:**
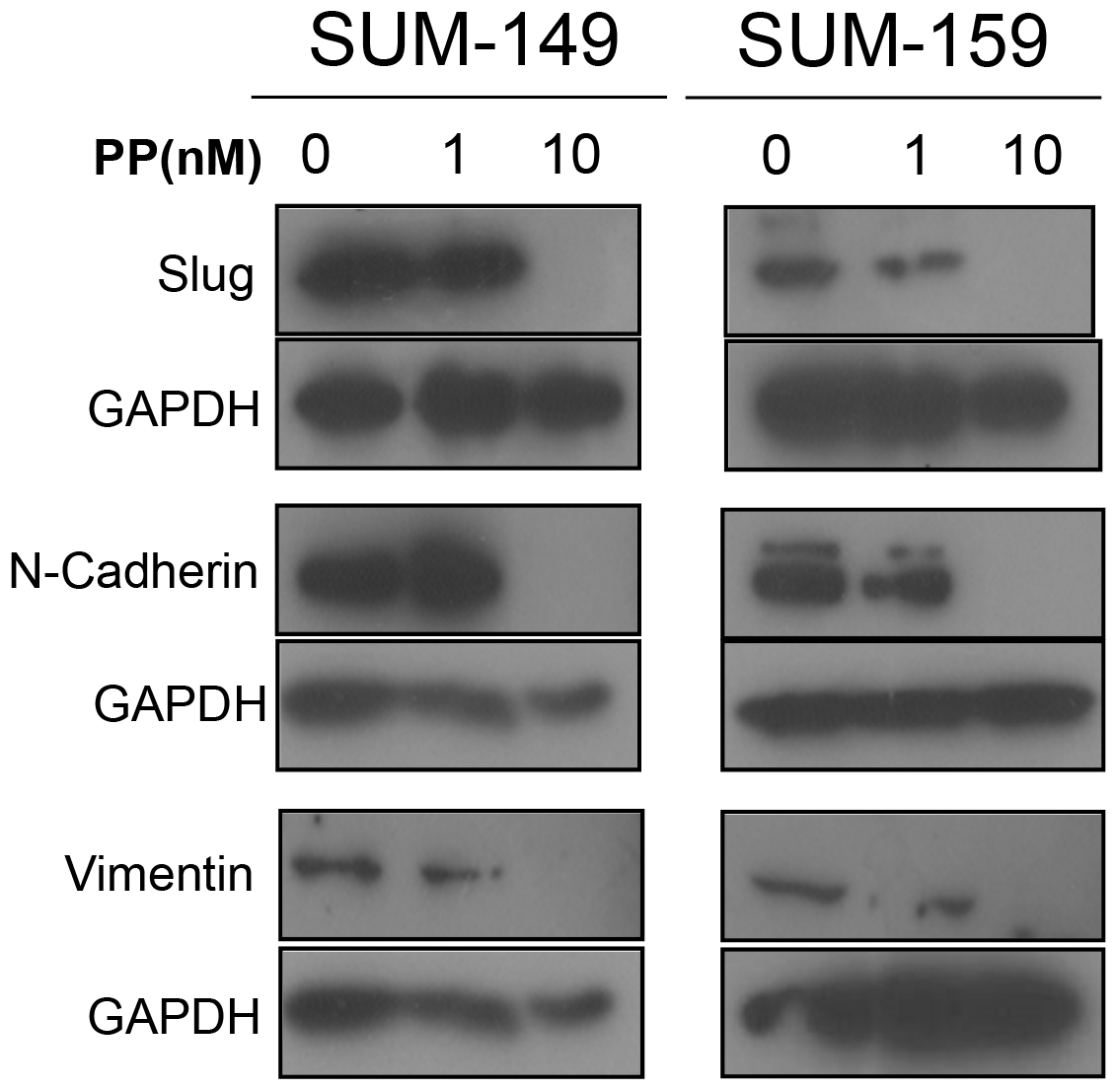
EMT markers were down-regulated in SUM-149 and SUM-159 cells treated with PP. SUM-149 and SUM-159 cells were treated with different doses of PP for 96 hours followed by Western blotting analysis using antibodies targeting different EMT markers. Slug, N-cadherin, and vimentin were down-regulated by 10 nM PP. The results shown are representative of three independent experiments.

Next, we investigated the mechanisms that may contribute to the effects of PP on breast cancer TIC surrogates. The EMT pathway is an important regulator of stem cell self-renewal [43], and WNT signaling has been reported to be necessary for EMT [44]. We examined whether EMT markers are down-regulated by PP in human breast cancer cells. As shown in Fig. 7, PP treatment down-regulated EMT-associated proteins N-cadherin, Slug, and vimentin.

### PP sensitized differentiated cancer cells, not TICs, to radiation therapy

**Figure 8:**
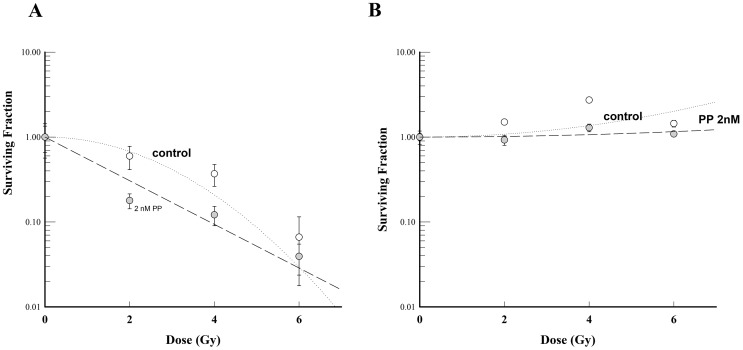
PP sensitized SUM-149 cells to radiation therapy. SUM-149 cells cultured in normal media were treated without or with 2 nM PP for 96 hours, followed by treatment with the indicated dose of radiation. Then cells were trypsinized and seeded in mammosphere media (A) or monolayer media (B). The results are representative of at least three independent experiments.

Because WNT signaling has been shown to play an important role in radiation resistance of breast cancer TICs [24], we tested whether PP sensitizes breast cancer TIC surrogates to radiation therapy using a monolayer-based clonogenic assay. Clonogenic assay is the standard approach for assessing resistance; it yields a count of single cells capable of replicating sufficiently to form a colony of at least 50 cells. Although plating efficiency in this assay is correlated to tumor initiation [45] and thought to capture radiation resistance of cancer progenitors, given this is done in standard culture media containing FBS, it is not expected that the most primitive cells maintained in mammosphere culture would be maintained or expanded into colonies in this assay system although stem cells have been thought to represent a subset of the clonogenic cell pool independent of the assay [46]. PP radiosensitized the SUM-149 cells in conventional adherent culture to radiation therapy at doses of 2 nM (Fig. 8A). To examine the radiosensitivity of mammospheres, the clonogenic assay was repeated using mammosphere culture conditions, presumably representing more primitive TICs [47]. Radiosensitizion of SUM-149 mammospheres was not observed at this dose (Fig. 8B).

## Discussion

Our studies demonstrate that PP efficiently targeted breast cancer TIC surrogates *in vitro* and *ex vivo* in cells derived from aggressive inflammatory and metaplastic breast cancer subtypes. Moreover, it caused tumor growth delay and necrosis in a SUM-159 orthotopic mouse model and trended toward decreased lung metastasis in a spontaneous metastasis model with activated Shh signaling. These data suggest targeting WNT signaling with PP may be an effective clinical strategy to improve outcomes for aggressive breast cancers if toxicity can be overcome.

Both IBC and metaplastic breast cancer are aggressive subtypes of breast cancer associated with poor prognosis and resistance to conventional therapies. Not surprisingly, both have been shown to be enriched in expression signatures derived from TICs, suggesting that TIC targeting may provide the most benefit for these subgroups of patients [48], [49]. The hallmark of IBC is skin involvement, which appears as erythema and edema (called peau d'orange) in the skin of the breast. Pathologically, dermal tumor emboli in the dermal lymphatics of the skin are evident [50]. Recently, dermal emboli of IBC were shown to have TIC properties, and mammospheres were suggested to recapitulate the dermal emboli of IBC [51]. In addition, ALDH1 expression in patients with IBC was associated with worse overall survival [52]. We speculate that efficient mammosphere/Aldefluor targeting of IBC cell lines will provide insight regarding treatment of IBC and aggressive breast cancer.

Numerous efforts have been made to identify compounds that specifically target TICs, and several compounds have been identified to target breast cancer TICs. In a study by Gupta et al., salinomycin, a potassium channel blocker used for treatment of Coccidia parasites in chickens, was identified to target breast cancer stem cells [29]. Li et al. have shown that the dietary supplement sulforaphane also targets breast cancer TICs and suggested that the mechanism for targeting breast cancer TICs involved WNT signaling [31]. WNT signaling has also been associated with aggressiveness of IBC. Genes downstream of the WNT signaling pathway were up-regulated in IBC patients with the ER-negative/Her2-negative subtype compared to non-IBC patients with the same subtype [53]. WNT signaling has also been implicated in the invasiveness and metastasis of breast cancer [54]. These studies support therapeutic efficacy of inhibiting WNT in aggressive breast cancer models.

PP is a quinoline-derived cyanine dye. In the 1950s, it was FDA approved for its antihelmintic properties [32]. It was also shown to have activities toward animal-like protists, such as *Plasmodium falciparum* and *Cryptosporidium parvum* [55], [56]. Recently, it was described that PP delays tumor growth in pancreatic [57], [58] and colon [58] cancer models. Although it has been used in the clinic for more than 50 years, the pharmacological mechanisms of PP only began to be elucidated in the last 10 years. Very recently, it was reported that PP inhibits WNT signaling via activation of casein kinase 1α (CK-1α) [34], [59]. WNT signaling inhibition *in vitro* was studied by examining the expression of WNT signaling-targeting genes, such as c-Myc, and the activity of TOP-luciferase, a functional assay [34]. *In vivo*, WNT signaling inhibition was assessed using the classic *Xenopus* secondary axis formation assay [34]. These studies demonstrated that CK-1α, a key kinase that phosphorylates β-catenin for degradation [60], is targeted directly and activated by PP. Although this report demonstrated selectivity compared to other kinases examined, other functions likely independent of WNT signaling have been described. PP is a non-competitive androgen receptor inhibitor [61], and unfolded protein response (UPR) signaling is one of the targets down-regulated by treatment with PP [58]. The NADH-fumarate reductase system, a metabolic system in mitochondria, was also found to be inhibited by PP [62]. Further studies using WNT inhibitors that are structurally distinct derivatives of PP may answer these questions.

In our studies, we used PP to test the role of WNT signaling in breast cancer TICs. In agreement with the work by Thorne et al. [34], we showed that PP efficiently down-regulates the expression of β-catenin, at the concentration of 10 nM in two different cancer cell lines. Furthermore, we used a TOP-GFP construct to test the inhibition activities of PP on WNT signaling in cancer cells and found that PP can inhibit WNT activities at a similar dose (Fig. 1B). In addition, we demonstrated that PP can target breast cancer TICs *in vitro*. Using the mammosphere formation assay and the ALDH assay, we observed that PP inhibited primary and secondary mammosphere formation at a dose below 10 nM. Such observation was further validated by *ex vivo* experiments using fresh patient samples from pleural fluid (Fig. 6). The dose used to inhibit mammosphere formation was 50 times smaller than the dose toxic to cells, suggesting selectivity against less differentiated cells. These data are in agreement with a previous report, in which blocking of WNT signaling decreased mammosphere formation, but had no toxic effect on cancer cells [54]. Therefore, the dose that inhibits breast cancer TICs *in vitro* is similar to the dose that inhibits WNT signaling.

We further examined the effect of PP *in vivo*, using an orthotopic model of SUM159 cells injected into the mammary glands of SCID/Beige mice. IP injections of PP delayed the tumor growth at 1 mg/kg. Previous studies that examined the effects of PP administered orally to mice confirm minimal absorption from the gut [33]. Moreover, a trend for decreased lung metastasis was observed in a murine Tumor505 model in which PP was administered IP.

In cancer therapy, it is important to eliminate not only the TICs but also the differentiated cancer cells. Therefore, combination therapies (TIC-killing agents plus debulking agents) to eliminate both types of cells are preferred. We tested the ability of PP to sensitize breast cancer cells to radiation therapy. We found that PP sensitized the cancer cells to radiation therapy in standard monolayer assays, but not in mammosphere clonogenic assays. Nevertheless, this is the first report that PP can sensitize tumor cells to radiation therapy. This might be a feasible topical approach for IBC to limit toxicity.

To elucidate the mechanism involved in the effects here described, we examined the expression of several proteins. Down-regulation of EMT affects the self-renewal of breast cancer TICs [43], and EMT has been shown to be a downstream target of WNT [63], [64]. Moreover, it has been reported that WNT-dependent expression of the EMT marker Slug has been associated with breast cancer invasiveness and metastasis [54]. Therefore, we believe that down-regulation of EMT by the inhibition of WNT signaling through PP may be the mechanism of TIC inhibition. Nevertheless, we acknowledge that other mechanisms or pathway cross-talk might be involved in the TIC-inhibition effect caused by PP. PP has been shown to have multiple activities besides inhibition of WNT signaling, including UPR inhibition [58], androgen receptor non-competitive inhibition [61], and NADH-fumarate reductase system inhibition. However, the direct targets of PP in those processes have not been identified and could be the effect of WNT signaling.

In summary, PP selectively killed breast cancer TICs and had excellent anti-tumor activity *in vivo* at 1 mg/kg. However, there are multiple obstacles before PP can be moved into clinical trials for cancer therapy. Major concerns will be its distribution and toxicity. Data regarding its absorption and distribution through the intravenous and IP delivery methods are not available. The difference here between the results from IP and IT delivery suggests that distribution of PP to the cancer is not efficient and a higher dose through IP delivery may be necessary. However, our experience and others' [61] suggested that 1 mg/kg is the highest dose mice can tolerate through IP delivery. So, improving distribution and delivering a low dose with low toxicity are a key step before PP can be tried in the clinic. The best way to address these issues may be to deliver PP by a nanovehicle or to use less toxic derivatives.

## Supporting Information

Figure S1 PP delayed tumor growth, caused tumor necrosis, and decreased the ALDH-positive population when it was administered intratumorally. SUM-159 cells were injected into the mammary fat pads of SCID/Beige mice. When tumor volumes approached approximately 300 mm^3^, either vehicle (DMSO) or 0.1 µg PP was injected into tumor directly. (A–C) PP delayed the tumor growth (A), caused tumor necrosis (B), and decreased the ALDH-positive population (C). (D) Histologic differences observed between necrotic tumor and control tumor. (E) The percentages of mice that developed necrosis after treatment.
(TIF)

Figure S2 PP might decreased WNT signaling *in vivo*. Nude mice implanted with SUM-159 cells transfected with 7-TFP construct were treated with PP (1 mg/kg) for 2 weeks. Luciferase signaling of one mouse was recorded before and after treatment (A). Data were summarized also summarized and plot (B).
(TIF)

Figure S3 PP might decrease lung metastasis in a mouse tumors 505 model. Mouse tumor 505 chunks were implanted into the mammary fat pads of SCID/Beige mice. Mice were treated with PP or DMSO alone. (A) Percentages of lung occupied by metastases for control and treated mice. (B) Histologic differences in the lungs of control and treated mice.
(TIF)
