# Supplementary figures and images for: The Antihelmintic Drug Pyrvinium Pamoate Targets Aggressive Breast Cancer
Source: PLoS One. 2013 Aug 27;8(8):e71508. doi: 10.1371/journal.pone.0071508 (PMC3754994; doi:10.1371/journal.pone.0071508)

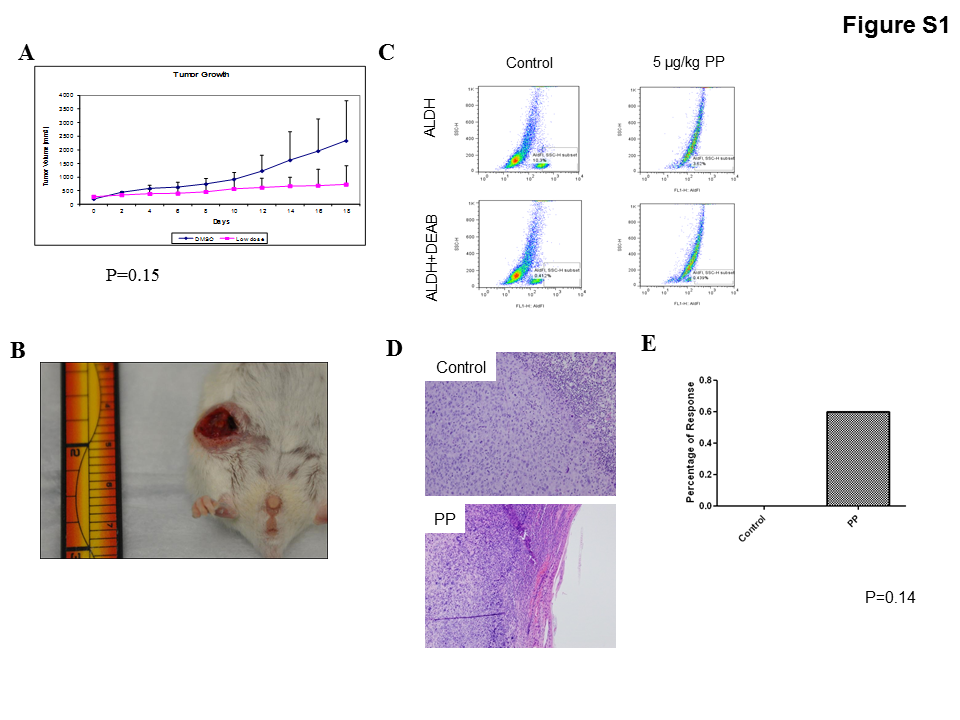

Supplement: Figure S1 — PP delayed tumor growth, caused tumor necrosis, and decreased the ALDH-positive population when it was administered intratumorally. SUM-159 cells were injected into the mammary fat pads of SCID/Beige mice. When tumor volumes approached approximately 300 mm3, either vehicle (DMSO) or 0.1 µg PP was injected into tumor directly. (A–C) PP delayed the tumor growth (A), caused tumor necrosis (B), and decreased the ALDH-positive population (C). (D) Histologic differences observed between necrotic tumor and control tumor. (E) The percentages of mice that developed necrosis after treatment. (TIF) [file pone.0071508.s001.tif]

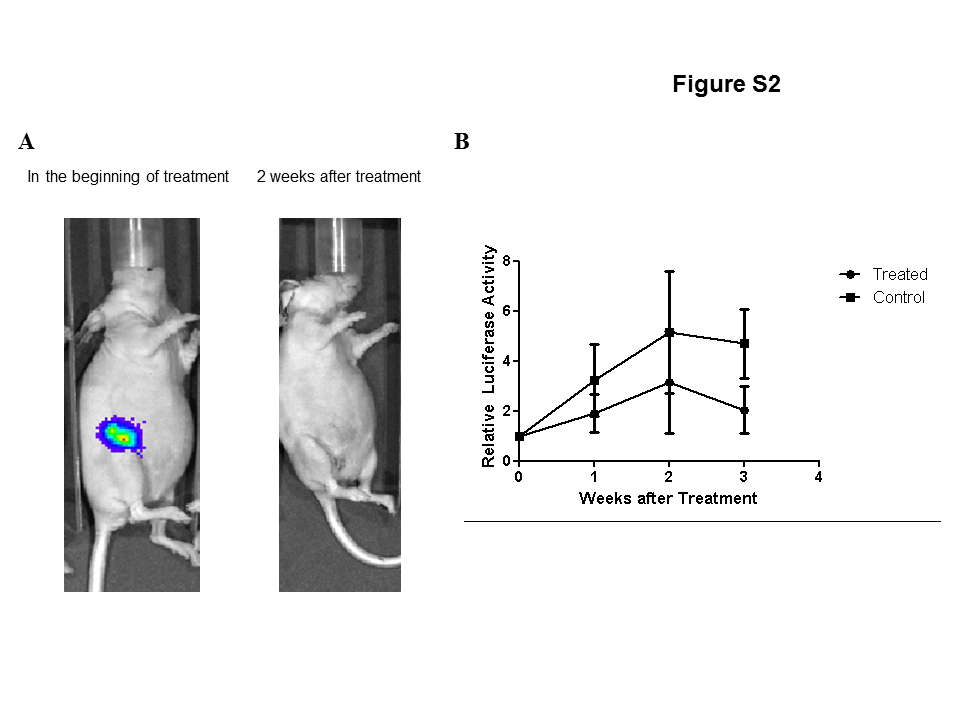

Supplement: Figure S2 — PP might decreased WNT signaling in vivo. Nude mice implanted with SUM-159 cells transfected with 7-TFP construct were treated with PP (1 mg/kg) for 2 weeks. Luciferase signaling of one mouse was recorded before and after treatment (A). Data were summarized also summarized and plot (B). (TIF) [file pone.0071508.s002.tif]

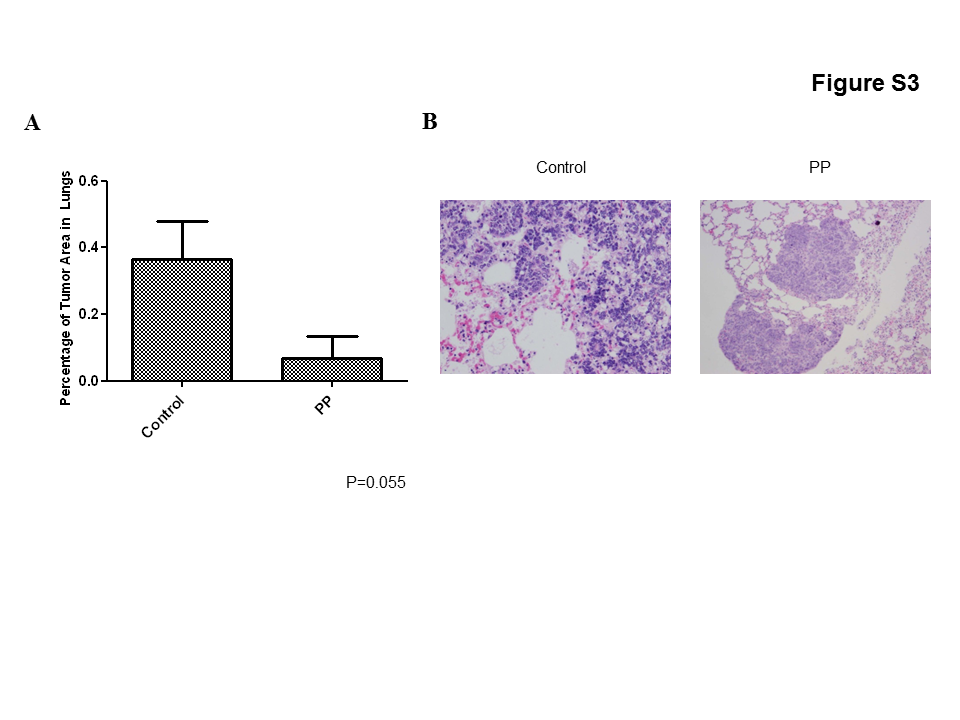

Supplement: Figure S3 — PP might decrease lung metastasis in a mouse tumors 505 model. Mouse tumor 505 chunks were implanted into the mammary fat pads of SCID/Beige mice. Mice were treated with PP or DMSO alone. (A) Percentages of lung occupied by metastases for control and treated mice. (B) Histologic differences in the lungs of control and treated mice. (TIF) [file pone.0071508.s003.tif]
